# The non-coding landscape of head and neck squamous cell carcinoma

**DOI:** 10.18632/oncotarget.9979

**Published:** 2016-06-13

**Authors:** Angela E. Zou, Hao Zheng, Maarouf A. Saad, Mehran Rahimy, Jonjei Ku, Selena Z. Kuo, Thomas K. Honda, Jessica Wang-Rodriguez, Yinan Xuan, Avinaash Korrapati, Vicky Yu, Pranav Singh, Jennifer R. Grandis, Charles C. King, Scott M. Lippman, Xiao Qi Wang, Andrew Hinton, Weg M. Ongkeko

**Affiliations:** ^1^ Department of Otolaryngology-Head and Neck Surgery, University of California San Diego, La Jolla, California, United States of America; ^2^ Veterans Administration Medical Center and Department of Pathology, University of California San Diego, La Jolla, California, United States of America; ^3^ Department of Otolaryngology-Head and Neck Surgery, University of California San Francisco, San Francisco, California, United States of America; ^4^ Department of Pediatrics, University of California San Diego, La Jolla, California, United States of America; ^5^ Department of Medicine and Moores Cancer Center, University of California San Diego, La Jolla, California, United States of America; ^6^ Department of Surgery, The University of Hong Kong, Pokfulam, Hong Kong, China

**Keywords:** head and neck cancer, non-coding RNA, RNA-sequencing, cancer transcriptomics

## Abstract

Head and neck squamous cell carcinoma (HNSCC) is an aggressive disease marked by frequent recurrence and metastasis and stagnant survival rates. To enhance molecular knowledge of HNSCC and define a non-coding RNA (ncRNA) landscape of the disease, we profiled the transcriptome-wide dysregulation of long non-coding RNA (lncRNA), microRNA (miRNA), and PIWI-interacting RNA (piRNA) using RNA-sequencing data from 422 HNSCC patients in The Cancer Genome Atlas (TCGA). 307 non-coding transcripts differentially expressed in HNSCC were significantly correlated with patient survival, and associated with mutations in *TP53*, *CDKN2A, CASP8, PRDM9,* and *FBXW7* and copy number variations in chromosomes 3, 5, 7, and 18. We also observed widespread ncRNA correlation to concurrent *TP53* and chromosome 3p loss, a compelling predictor of poor prognosis in HNSCCs. Three selected ncRNAs were additionally associated with tumor stage, HPV status, and other clinical characteristics, and modulation of their expression *in vitro* reveals differential regulation of genes involved in epithelial-mesenchymal transition and apoptotic response. This comprehensive characterization of the HNSCC non-coding transcriptome introduces new layers of understanding for the disease, and nominates a novel panel of transcripts with potential utility as prognostic markers or therapeutic targets.

## INTRODUCTION

HNSCC is the sixth leading cancer worldwide, with an estimated 600,000 new cases annually and a 50% five-year mortality rate [1]. The molecular heterogeneity and diverse etiology of head and neck tumors remain prohibitive barriers to the development of improved treatments and prognostic tools. Previous characterization of molecular features in HNSCC [2–5], particularly with the aid of large-scale cancer genomics initiatives such as The Cancer Genome Atlas (TCGA) [6, 7], have generated important insights for stratifying HNSCCs and delineating tumor subtypes. However, these analyses, almost exclusively concentrated on the protein-coding genome, critically overlook alterations in the vast non-coding transcriptome that may substantially contribute to HNSCC pathogenesis and progression.

microRNAs (miRNAs) are the only class of non-coding RNAs (ncRNAs) relatively well-investigated in HNSCC, with previous studies profiling their dysregulation [8–10], association with patient survival [11, 12], and involvement in apoptotic and oncogenic pathways [13, 14]. Long non-coding RNAs (lncRNAs) have also been increasingly implicated in tumor biology, yet beyond studies of previously cancer-associated transcripts such as *HOTAIR*, *MALAT-1*, and *NEAT-1*, their significance in HNSCC is sparsely characterized [15–17]. The involvement of PIWI-interacting RNAs (piRNA) remains an even greater enigma, its dysregulation in breast, lung, and gastric cancers having only recently been uncovered [18].

Preliminary investigation of lncRNAs in HNSCC, coupled with the recent implication of piRNAs in cancers, highlights the need to systematically characterize ncRNA expression and functional significance in the HNSCC tumor population. Utilizing next-generation sequencing and clinical data from 422 HNSCC patients in TCGA, we profiled the global expression patterns and dysregulation of all three ncRNA classes in HNSCC. We sought to not only generate insights into the altered transcriptomic landscape of HNSCC, but also integrate novel findings with established molecular pathways in HNSCC pathogenesis and progression and with differences in tumor phenotype and patient outcome.

## RESULTS

### Non-coding RNA expression and dysregulation in HNSCC

**Figure 1:**
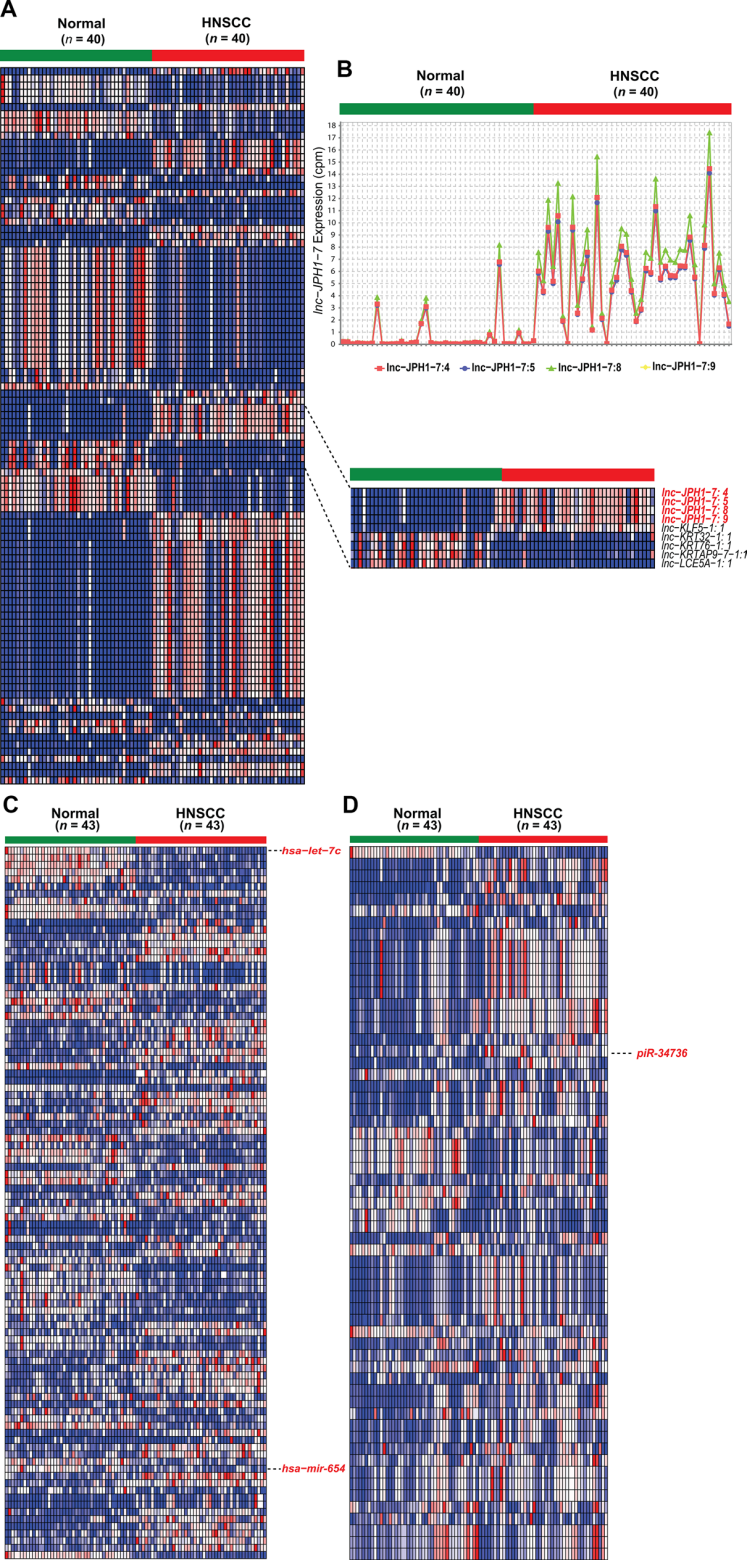
Heatmaps of significantly differentially expressed non-coding RNAs in HNSCC (**A**) Heatmap depicting normalized expression levels (in counts-per-million) of the 100 lincRNA transcripts with the largest magnitude of dysregulation in HNSCCs compared to paired normal samples (*FDR* < 0.0001). Inset highlights the 4 experimentally validated isoforms of *lnc-JPH1-7*. (**B**) Expression plot of *lnc-JPH1-7* in HNSCCs compared to adjacent normal tissue. (**C–D**) Heatmaps depicting normalized expression levels (in counts-per-million) of (D) 232 miRNAs (*FDR* < 0.05) and (E) 61 piRNAs (*FDR* < 0.05) dysregulated in HNSCC tumors, highlighting discussed or ultimately experimentally validated transcripts.

Demographics and clinical characteristics for the total HNSCC cohort used in the study are provided in Supplementary Table 1. To identify HNSCC-dysregulated ncRNAs, we utilized all HNSCC tumor-paired adjacent normal RNA-sequencing data in TCGA on 15 June 2014. These data consisted of: (1) 40 tumor-normal RNA-seq datasets, for lncRNA profiling; and (2) 43 tumor-normal small RNA-seq datasets and Level 3 gene expression analyses, for piRNA and miRNA profiling, respectively (dataset IDs in Supplementary Dataset 1). ncRNA expression was obtained by alignment of sequencing datasets to custom gene annotations containing 113,438 lncRNA transcripts and 27,127 piRNAs, or extracted from corresponding TCGA Level 3 gene expression analyses profiling 1,046 miRNAs. Using negative binomial-based differential expression testing, we identified 9,681 lncRNA transcripts, 232 miRNAs, and 61 piRNAs significantly dysregulated (*FDR* < 0.05) in HNSCCs relative to paired normal tissue. We focused subsequent study on 596 intergenic lncRNAs (lincRNAs) exhibiting reliable expression and ≥ 4-fold dysregulation in HNSCCs (*FDR* < 0.0001) (Supplementary Dataset 2, Figure 1A–1B), while retaining all differentially expressed miRNAs and piRNAs as candidates (Supplementary Datasets 3–4; Figure 1C–1D).

Our analysis confirmed the previously-reported dysregulation of several miRNAs in HNSCC, including downregulation of *miR-375* [14], the *miR-29* family [19]*, miR-204* [20], and *miR-99* [21], and upregulation of *miR-196* [22], *miR-21* [23], and *miR-31* [23, 24]. Our results also revealed miRNAs with unexplored roles in the context of HNSCC, including *miR-654,* previously associated with prostate metastasis [25] and hepatocellular carcinoma [26], and *let-7c*, encoded in a genomic cluster distinct from *let-7* miRNAs harboring documented links to HNSCC [10, 27] (Figure 1c).

Among lncRNAs, we confirm previously-reported downregulation of *GAS5* and *MEG-3* [28], and identify for the first time dysregulation of cancer-associated *H19* and *PCAT-1* in HNSCCs. However, we observed only modest (< 4-fold) alterations in the expression of all four transcripts relative to normal (Supplementary Dataset 5). Furthermore, many other established, cancer-linked lncRNAs, including *HOTAIR, MALAT-1, NEAT-1,* and *UCA-1*, were found to be either extremely lowly expressed (mean and median cpm < 1) or insufficiently dysregulated in HNSCCs (Supplementary Dataset 5). Meanwhile, comparison of the HNSCC-dysregulated piRNAs to preliminary studies of their expression in other cancers yielded similarly sparse overlap, with only *piR-34736* and *piR-36318* previously identified in breast cancer [18].

### Identification of dysregulated non-coding RNAs correlated to patient survival

**Figure 2:**
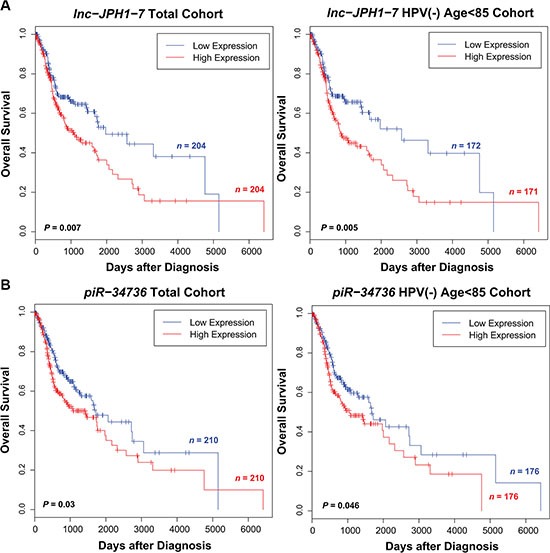
Selected ncRNAs exhibiting significant correlation to HNSCC patient survival (**A–B**) Kaplan-Meier curves showing survival outcomes according to relative high and low expression of (A) *lnc-JPH1-7*, (B) *piR-34736* in HNSCC tumors. Association of these ncRNAs with patient survival is significant in both the full tumor cohort and among the subset of HPV-negative, age < 85 patients.

After obtaining ncRNA expression in all remaining TCGA HNSCCs with clinical data (dataset IDs in Supplementary Dataset 6), we screened each ncRNA class for transcripts significantly associated with patient survival. Because patient age and HPV status are observed to produce distinct survival outcomes in HNSCC patients [1], we limited the cohorts for our initial screens to HPV- negative patients < 85 years of age. We next evaluated the ncRNAs for prognostic potential among all patients regardless of age or HPV status. Under univariate and multivariate Cox regression analyses in both cohorts, we identified 276 intergenic lncRNAs, 21 miRNAs, and 6 piRNAs significantly predictive of overall patient outcome (Supplementary Datasets 7–9). 2 ncRNAs ultimately selected for experimental validation exhibited prognostic significance both among HPV-negative, age < 85 patients and in the full tumor cohort (Figure 2).

### Association of non-coding RNAs with known HNSCC genomic alterations

**Figure 3:**
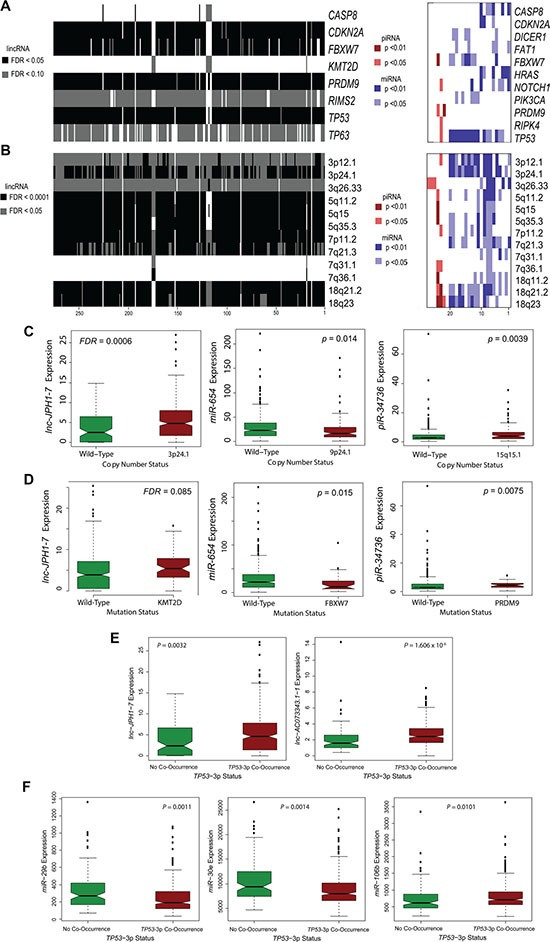
Association of prognostic ncRNAs with somatic mutations and copy number variations in HNSCCs (**A**) Heatmap showing widespread correlation between ncRNA expression level and select somatic mutations of high incidence in HNSCC tumors. ncRNAs are arranged alphabetically, with numeric position on heatmap indicated in source data. (**B**) Heatmap showing abundant correlations between ncRNA expression level and common CNVs in HNSCCs. ncRNAs are arranged alphabetically, with numeric position on heatmap indicated in Supplementary Dataset 10. (**C–D**) Notched boxplots depicting specific (C) ncRNA-CNV correlations and (D) ncRNA-mutation correlations for selected, survival-associated ncRNAs (Wilcoxon rank-sum, *P <* 0.05 for miRNA and piRNA; *FDR* < 0.1 for lincRNAs). (**E–F**) Notched boxplots depicting specific (E) lincRNAs and (F) miRNAs exhibiting significant correlation in expression to concurrent loss of TP53 and 3p in HNSCCs (Wilcoxon rank sum, *P* < 0.05).

Multiple studies have identified compelling patterns of co-occurrence and synergistic interaction among genomic and molecular alterations in cancer [6, 29–32]. To locate the potential functions of survival-associated ncRNAs in relationship to canonical HNSCC driver events, we employed Wilcoxon rank-sum testing to identify correlations between ncRNA expression and tumor mutational status or copy number variation. TCGA HNSCC mutation and copy number calls were obtained from the Broad Institute GDAC Firehose, with attention restricted to 26 frequently occurring somatic mutations in HNSCCs [4] and 73 copy number alterations. Notably, expression levels of many ncRNAs were strongly correlated to *TP53* mutation, as observed in 256 among 276 survival-associated lincRNA transcripts (*FDR* < 0.0001), 12 among 21 prognostic miRNAs (*p* < 0.01), and 1 among 6 survival-associated piRNAs (*p* < 0.05) (Figure 3A). We also show frequent ncRNA association with mutations in *CASP8, CDKN2A*, histone methyltransferase *PRDM9*, and cyclin E, Notch, and c-Myc regulator *FBXW7* (Supplementary Datasets 11– 12, Figure 3A). Additionally, pairwise analyses between ncRNA expression and incidence of copy number variations revealed widespread correlations with 3p, 5p, 7p, and 18q deletion, and 3q and 7q amplification (Supplementary Datasets 13–14, Figure 3B).

A recent study found that HNSCCs containing both *TP53* mutation and deletions on the chromosome 3p arm were dramatically associated with advanced tumor stage and poor clinical outcomes [6]. To explore the potential implications and molecular basis underlying this phenomenon, we further assessed the relationship between expression of our candidate ncRNAs and incidence of *TP53*-3p events. The high-risk *TP53-*3p cohort consisted of: (1) HNSCCs harboring both *TP53* mutation and 3p deletion, and (2) all HPV-positive HNSCCs (which exhibit *TP53* inactivation by HPV) harboring 3p deletion.

In total, 269 of 276 survival-correlated lincRNAs (*FDR* < 0.05) and 11 prognostic miRNAs (*p* < 0.05), were significantly associated with *TP53-*3p co-occurrence (Supplementary Dataset 15). Among these were a number of miRNAs previously characterized in HNSCC, notably *miR-29b-1, -2* [33], *miR-370* [34], *miR-126* [35],*miR-337* [36]*, miR-30e* [37], and *miR-106b* [9]. Taken together, these findings suggest a substantial role for our identified ncRNAs in key oncogenic pathways and molecular signatures.

### qRT-PCR validation and characterization of non-coding RNAs in HNSCC cell lines

Having attained a global perspective of the HNSCC non-coding transcriptome, we undertook a focused investigation of several ncRNAs by further evaluating their relationship to clinical features and their significance *in vitro*. One transcript from each ncRNA class was chosen for functional study: *lnc-JPH1-7* (lncRNA), *miR-654-3p* (miRNA), and *piR-34736* (piRNA). *miR-654-3p* was selected based on its novel identification as a differentially expressed miRNA in HNSCCs, while *lnc-JPH1-7* and *piR-34736* were selected based on novel dysregulation in HNSCC, as well as significant association with survival among both HPV-negative and HPV-positive patients (Figure 2). Dysregulation of the selected ncRNAs was verified by qRT-PCR using 2 normal oral epithelial cell lines compared to 5–6 established HNSCC cell lines (Supplementary Figure S2).

**Figure 4:**
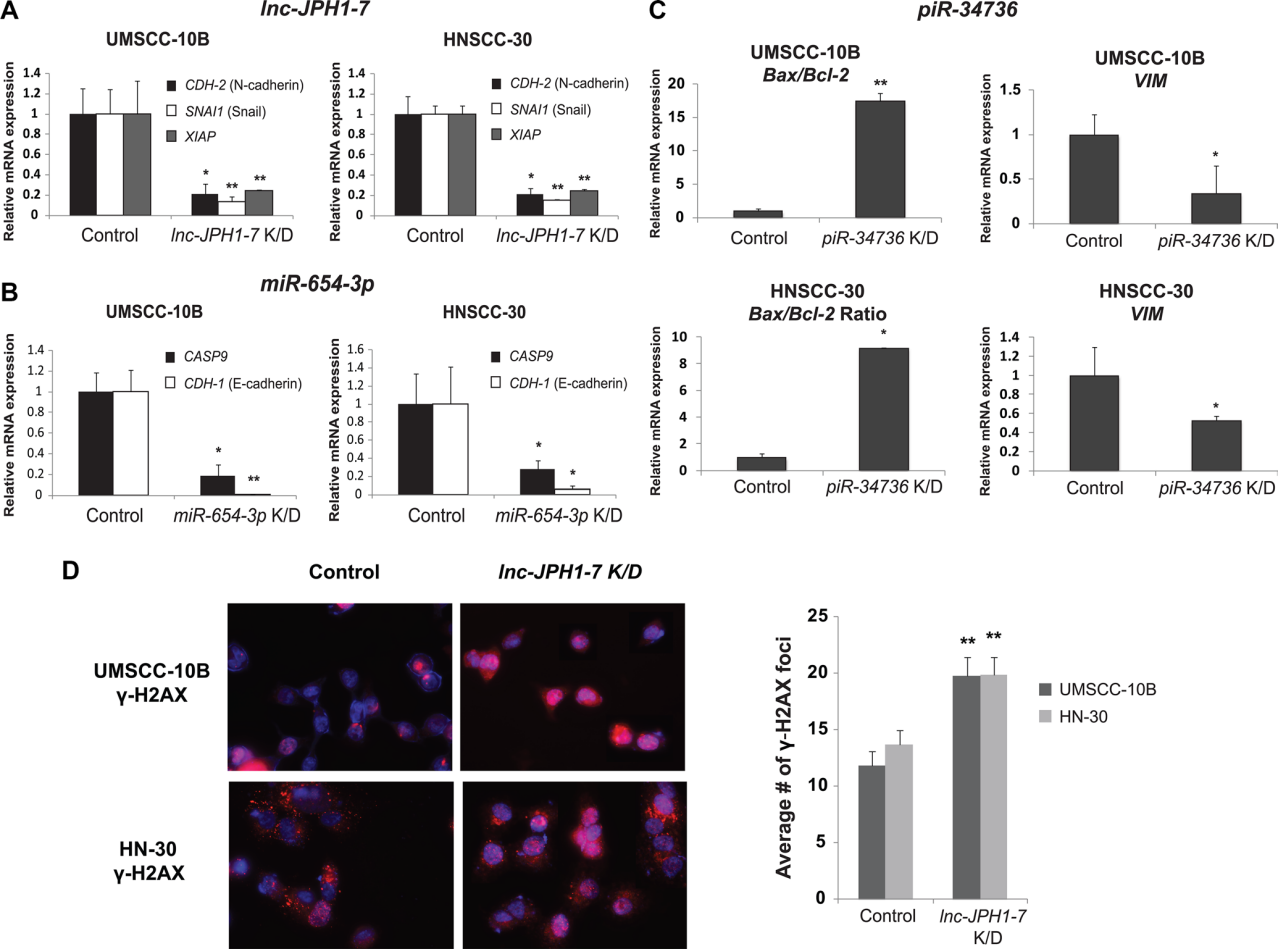
Effects of *in vitro lnc-JPH1-7, miR-654-3p, and piR-34736* manipulation on expression of EMT and apoptosis genes (**A–C**) Knockdown of *lnc-JPH1-7, miR-654-3p,* and *piR-34736* led to the repression of EMT and anti-apoptotic genes in HNSCC cell lines (*n* = 3). *p < 0.05, **p < 0.01, ***p < 0.001, Student's *t*-test. (**D**) Immunofluorescence images showing increased expression of γ-H2AX, and significantly increased accumulation of γ-H2AX foci in UMSCC-10B and HN-30, following *lnc-JPH1-7* knockdown.

*lnc-JPH1-7*, a 1,164-nt transcript located on chromosome 8q21, is upregulated by 35-fold in TCGA HNSCCs. We found that elevated *lnc-JPH1-7* levels in HNSCCs associated not only with poor prognosis, methyltransferase *KMT2D* (*MLL1*) mutation, and concurrent *TP53* mutation and 3p deletion, but also significantly correlated to advanced tumor stage (*p* = 0.02; Supplementary Figure S3a). Furthermore, higher median *lnc-JPH1-7* expression was observed among HPV-negative patients and patients reporting history of smoking (Supplementary Figure S3B-S3C). We subsequently manipulated the expression of *lnc-JPH1-7 in vitro* to evaluate its oncogenic characteristics. shRNA-mediated knockdown of *lnc-JPH1-7* reduced the expression of EMT-promoting genes for N-cadherin (*CDH-2*) and Snail (*SNAI1*), as well as anti-apoptotic gene *XIAP*, in HNSCC cell lines UMSCC-10B and HN-30 (Figure 4A). Next we assessed the effects of *lnc-JPH1-7* on apoptotic DNA fragmentation using the DNA double-strand break and apoptosis marker γ-H2AX. γ-H2AX accumulates in early apoptosis with the onset of DNA fragmentation, as a direct consequence of caspase activation [38]. Immunofluorescence showed significant upregulation of γ-H2AX in *lnc-JPH1-7*-knockdown cell lines, with many cells exhibiting pan-nuclear γ-H2AX staining, a defining characteristic of the apoptotic γ-H2AX response (Figure 4D) [39, 40]. We also observed significantly increased punctate γ-H2AX foci in non-pan-nuclear staining cells with manipulated *lnc-JPH1-7* expression (Figure 4D).

*miR-654-3p* is downregulated by 2-fold in TCGA HNSCCs, with lower expression in tumors associated with increased incidence of *FBXW7* mutation (Figure 3A, 3D). Although implicated in other malignancies, most notably in prostate cancer as a regulator of apoptosis and invasion [25], underexpression of *miR-654-3p* has not been previously identified in HNSCC. We found that median *miR-654-3p* levels are reduced in advanced-stage HNSCCs compared with early-stage tumors (Supplementary Figure S4A). Additionally, further knockdown of *miR- 654-3p* expression in HNSCC cell lines UMSCC-10B and HN-30 led to the repression of E-cadherin and Caspase-9 gene expression (Figure 4B). Finally, *piR-34736* (DQ596670), upregulated by 2-fold in TCGA HNSCCs and correlated to patient survival and *PRDM9* mutation (Figure 3A, 3D), was recently observed to be downregulated in breast cancers, with uncharacterized functional significance [18]. To investigate the role of *piR- 34736* in HNSCC malignancy, we suppressed *piR- 34736* expression in the UMSCC- 10B and HN-30 cell lines, resulting in significantly increased *Bax/Bcl2* ratio and repression of EMT-mediator Vimentin (*VIM*) (Figure 4C). We also observed significant association between elevated *piR-34736* expression and advanced tumor stage (*p <* 0.005; Supplementary Figure S4B), and increased median *piR-34736* expression with tumor grade and recent smoking (Supplementary Figure S4C).

## DISCUSSION

The recent implication of ncRNAs in a vast array of biological functions has established the non-coding transcriptome as a new paradigm in understanding and subverting disease processes. For malignancies such as HNSCC in which traditional genomic characterizations have produced minimal improvements in patient outcome, ncRNAs may represent a particularly promising avenue for novel diagnostic and therapeutic strategies. Here, we comprehensively analyzed the expression patterns of three classes of ncRNAs in HNSCC using RNA-sequencing data from 422 TCGA HNSCC cases. Among the vast number of significantly differentially expressed miRNAs, lncRNAs, and piRNAs, we ultimately identified 303 transcripts demonstrating both dysregulation in HNSCC and association with patient survival, most of which have not been previously linked to cancer pathogenesis and progression. Notably, many lncRNAs harboring well-documented links to other cancers were found to exhibit low expression or limited dysregulation in HNSCC. For instance, tumor suppressors *GAS5* and *MEG-3* were downregulated by less than 2-fold in tumors (Supplementary Dataset 5). *HOTAIR*, although reported to be upregulated in multiple malignancies, including head and neck tumors from certain anatomic sites [15, 16], was shown by our RNA-sequencing analysis to be expressed at very low levels (median cpm < 0.5) in HNSCCs overall. Similarly, our panel of dysregulated and survival-correlated piRNAs was largely distinct from those previously identified in lung, gastric [41], and breast [18] cancers. These findings underscore the importance of using unbiased sequencing approaches to globally evaluate ncRNAs in terms of both reliable expression and significant dysregulation. The limited applicability of previously identified cancer-associated ncRNAs to HNSCC acutely highlights the necessity of continuing to identify novel non-coding transcripts exhibiting both functional relevance and therapeutic potential in HNSCC.

One notable challenge of studying ncRNAs is determining their precise contributions to disease pathogenesis and progression. To generate insight into the potential functions of our identified ncRNAs, we examined their expression in tumors in relationship to HNSCC genomic signatures, and observed a particularly compelling link between many ncRNAs and incidence of *TP53* mutation. Several miRNAs and lncRNAs have been previously characterized in p53 regulatory networks [42, 43], including the p53-induced *miR-34* family [44] and *lincRNA-p21* [45], both of which facilitate large-scale transcriptional repression of genes in the p53 pathway. Thus, the discovery of abundant p53-ncRNA associations in HNSCC, while underscoring the widespread molecular alterations resulting from *TP53* loss alone, may also implicate many additional non-coding transcripts as active regulators of the p53 response or as mediators of its downstream effects. While p53-ncRNA networks represent a growing field of study, the nature of ncRNA interactions with other somatic mutations and genomic alterations has remained virtually unexplored and unevaluated. Extensive ncRNA correlations with *CASP8, CDKN2A, PRDM9,* and *FBXW7* mutations, and with chromosome 3, 5, 7, and 18 copy number variations, suggest that the tumorigenic significance of these aberrations is administered at least in part by ncRNA activity. Functional investigation of these associations may yield valuable insights into how malignant transformation in general is facilitated through genomic and non-coding transcriptomic dependence. Specifically, the ncRNAs we have identified to be correlated with concurrent *TP53* mutation and 3p deletion may be involved in mediating the mechanisms for their co-occurrence in HNSCC and other malignancies [6] and for their synergistic tumorigenic effects.

To substantiate the functional potential of our ncRNAs, we manipulated the expression of several transcripts in HNSCC cell lines and observed their resulting impact on key cancer-associated genes. All three ncRNAs selected for study were found to influence expression of genes involved in EMT and apoptosis, underscoring the importance of these pathways in HNSCC, and linking the prognostic significance of these ncRNAs to the regulation of migratory and invasive characteristics in HNSCCs. *miR-654* exhibited negative regulation of E-cadherin and Caspase 9 in HNSCC cell lines, consistent with its proposed role of inhibiting prostate metastasis [25]. Meanwhile, although *piR-34736* was found to be underexpressed in breast cancers [18] and *lnc-JPH1-7* was also identified in esophageal (*ESCCAL-1*) [46] and lung (*LCAL80*) [47] squamous cell carcinomas, their clinical and molecular significance has, up to now, remained uninvestigated. Here, we show that both ncRNAs may exert distinctively oncogenic functions, their increasing expression in HNSCCs correlated to both significantly reduced survival and advanced tumor stage, and their knockdown in HNSCC cell lines leading to the repression of EMT-promoting and anti-apoptotic genes. Moreover, suppression of *lnc-JPH1-7* was also associated with accumulation of DNA double-strand break and apoptosis-associated γ-H2AX. Taken together, these results highlight the potential functional significance of our additional candidate ncRNAs, and underscore the relevance of all three ncRNA classes in tumorigenesis and metastasis.

Utilizing the comprehensive TCGA collection of next-generation sequencing and clinical data, we identified a panel of HNSCC-dysregulated ncRNAs correlated with patient survival, and characterized three transcripts *in vitro* to establish their roles in HNSCC pathogenesis and progression. Our results, while underscoring the vast array of ncRNAs whose links to HNSCC remain novel and unexplored, show that these transcripts are often involved in, and may significantly enhance, our understanding of molecular interplay even in the most central, well-studied, disease pathways and processes. By profiling the HNSCC non-coding transcriptome with unprecedented depth and scope and integrating its characteristics with established clinical and molecular features, this study provides a foundation for future endeavors to investigate ncRNAs in HNSCCs as well as other malignancies.

## MATERIALS AND METHODS

### TCGA RNA-sequencing and gene expression datasets

MapSplice-aligned TCGA BAM files were obtained from the UCSC Cancer Genomics Hub (https://cghub.ucsc.edu/) on 15 June 2014 for paired tumor and adjacent normal pairs, and on 1 August 2014 for unpaired tumors. To investigate lncRNA expression, we downloaded RNA-seq datasets for 40 tumor-adjacent normal pairs and 368 additional unpaired tumors, and to investigate piRNA expression, we downloaded small RNA-seq datasets for 43 tumor-adjacent normal pairs and 380 additional unpaired tumors.

miRNA expression data were derived from TCGA Level 3 normalized gene expression datasets (https://tcga-data.nci.nih.gov/tcga/findArchives.htm). To profile miRNAs, we downloaded miRNA-seq Level 3 analyses for the same cohort as used for piRNA expression profiling. The TCGA barcodes for all patients whose datasets were used in this study are provided in Supplementary Datasets 1 and 6.

### Non-coding RNA expression analysis

miRNA read counts were extracted from the TCGA Level 3 gene expression analyses. lncRNA and piRNA read counts were generated from sequencing datasets via BEDtools coverageBed (https://github.com/arq5x/bedtools2) [48] using lncRNA and piRNA annotation files. A lncRNA BED file containing 113,438 transcripts was obtained from LNCipedia 3.0 (http://lncipedia.org/) [49], a database curating lncRNAs from sources including the Broad Institute, Ensembl, Gencode, Refseq, and NONCODE. A BED file comprising 27,127 piRNAs was created by extracting all human piRNAs from the NONCODE v4.0 non-coding RNA database (http://www.noncode.org/) [50].

Read count tables were imported into edgeR v3.0 (http://www.bioconductor.org/packages/release/bioc/html/edgeR.html) [51], and lowly expressed ncRNAs (counts-per-million< 1in more than 50% of samples) were filtered from the analysis. Following TMM normalization, matched pair designs were applied to identify significantly differentially expressed ncRNAs between HNSCC and adjacent normal tissue.

All miRNAs and piRNAs identified as differentially expressed in edgeR were retained as candidates. To identify dysregulated intergenic lncRNAs, we downloaded a comprehensive annotation of protein-coding genes from UCSC (http://genome.ucsc.edu/cgi-bin/hgTables) and removed all lncRNAs overlapping protein-coding exons. We further filtered candidate lncRNA transcripts based on cutoffs for fold change magnitude (≥ 4 between HNSCC and normal) and false discovery rate (FDR) ≤ 10^−4^.

### Clinical data

Clinical data for 422 HNSCC cases were downloaded from the TCGA Data Portal on 1 August 2014 (https://tcga-data.nci.nih.gov/tcga/findArchives.htm). Reported patient outcomes are based on all-cause mortality. Patient HPV status was obtained from sequencing datasets archived on cBioPortal (http://www.cbioportal.org/public/) by the TCGA HNSCC analysis working group. For patients where sequencing-based HPV calls were unavailable, results from PCR-based MassARRAY HPV detection assays and HPV in-situ hybridization assays were retrieved from the TCGA Data Portal.

### Association of ncRNA expression with patient survival

Survival analyses were performed using Cox proportional hazards models, with ncRNA expression in tumors established as a binary variable based on expression above or below the median. Because patient age and HPV status have been shown to profoundly influence molecular signatures and clinical outcomes in HNSCC, we limited our initial cohort to HPV-negative patients less than 85 years of age, resulting in 343 for lincRNA survival analysis and 352 for miRNA and piRNA analyses (Supplementary Dataset 6). In our multivariate analysis, patient age was introduced as both a continuous and binary (age < or > = 75) to capture non-linear relationships between patient age and survival. *P*-values generated under this model for lincRNAs were corrected for multiple-hypothesis testing using the Benjamini-Hochberg false discovery rate correction, with a significance threshold of *FDR* < 0.05. ncRNAs ultimately selected for experimental validation were also evaluated under Cox analysis among all patients, regardless of age or HPV status.

### Association of ncRNA expression with tumor mutations and copy number aberrations

Mutation calls for the TCGA tumors were obtained from mutation annotation files (maf) generated by the Broad Institute GDAC Firehose on 5 September 2014. We focused our analysis on 26 most frequently mutated genes in HNSCCs, as determined by whole exome sequencing of an independent tumor cohort by Stransky et al. [4]. A Wilcoxon rank sum test was employed to test for significant associations between ncRNA expression level (counts-per-million) and mutational status. *P*-values among lincRNAs were corrected for multiple comparisons testing using the Benjamini-Hochberg procedure.

Copy number variations for the TCGA tumors were obtained from the GISTIC2 pipeline in Firehose on 24 September 2014. Similarly, all 73 significant (99% confidence) focal amplifications and deletions were analyzed for correlation to ncRNA expression level using Wilcoxon rank sum tests, followed by Benjamini-Hochberg correction of lincRNA *p*-values.

### Association of ncRNA expression with tumor stage, grade, invasion, and patient HPV and smoking status

Correlations between ncRNAs selected for experimental validation and clinical features were performed using the Kruskal-Wallis test. To investigate ncRNA association with HPV status, clinical data and ncRNA expression data (cpm) from the full tumor cohorts were used.

Analyses of ncRNA in relationship to tumor stage and grade, perineural invasion, and smoking were performed using the HPV-negative, age<85 cohorts. Patients with no available information for a given characteristic were filtered from analyses involving that variable.

In tumor stage analyses, patients with Stage IVA, B, and C tumors were classified as “Stage IV.” In smoking status analyses, patients listed as “Current smoker” or “Current reformed smoker for < or = 15 years” were classified as “Recent Smokers,” while patients listed as “Current reformed smoker for > 15 years” were classified as “Reformed Smokers.”

### Cell culture

The non-cancerous oral epithelial cell lines OKF4, OKF6, HOK were gifts from the Rheinwald Lab at Harvard Medical School. They were cultured in Keratinocyte-SFM(1X) with L-glutamine, supplemented with 0.2 ng/mL human recombinant epidermal growth factor (EGF), 25 ug/mL bovine pituitary extract (BPE), 0.3 mM calcium chloride, and penicillin streptomycin. Upon attaining 30% confluency, they were cultured in equal parts supplemented Keratinocyte-SFM medium and DFK medium. DFK was made with equal parts DMEM and F-12 and supplemented with 0.2 ng/mL EGF, 25 ug/mL BPE, 2mM L-glutamine, and penicillin streptomycin.

Established HNSCC cell lines used in this study were UMSCC-10B (larynx), UMSCC-22B (larynx), HN-1 (pharynx), HN-12 (tongue), and HN-30 (pharynx), SCC-4 (tongue) and Cal-27 (tongue). UMSCC-10B and UMSCC-22B were gifts from Dr. Tom Carey, University of Michigan, and HN-1, HN-12, and HN-30 were gifts from Dr. J.S. Gutkind, National Institute for Dental and Craniofacial Research. SCC-4 and Cal-27 were purchased from the American Type Culture Collection. Cell lines were cultured in DMEM supplemented with 10% fetal bovine serum (FBS), 2% streptomycin sulfate, and 2% L-glutamine, and incubated at 37°C in 5% CO_2_ and 21% O_2_.

### siRNA-mediated knockdown of *lnc-JPH1-7*

Select siRNA oligonucleotides targeting *lnc-JPH1-7* were custom synthesized (Life Technologies) with the following sequences: siRNA 1: GCAUGUAUU UUGGUCAAUATT siRNA 2: GAAGUUUAGUAAACC AAAATT.

HNSCC cell lines were transfected with a 100 pmol pool containing both *lnc-JPH1-7* siRNAs or with a control scramble sequence, using Lipofectamine 2000 (Life Technologies) per manufacturer's instructions. Transfection efficiency for the siRNAs was determined using qRT-PCR (Supplementary Figure S5), with total RNA extraction performed 48–72 hours following transfection.

### shRNA-mediated knockdown of *miR-654* and *piR-34736*

shRNA sequences targeting *miR-654* and *piR-34736* (listed in Supplementary Table 2) were PCR-amplified and directionally cloned into the pmiRZIP lentivector (SBI) at the *BamH*1/*EcoR*1 site. The inserts were confirmed using Sanger sequencing (GENEWIZ).

HNSCC cell lines were transiently transfected with the miRZIP constructs using Lipofectamine 2000 (Invitrogen), with empty vector used as a control. Transfection efficiency for the plasmids was determined using qRT-PCR (Supplementary Figure S5), with total RNA extraction performed 48–72 hours following transfection.

### RNA isolation and cDNA synthesis

Total RNA was extracted using the SurePrep TrueTotal RNA Purification Kit (Fisher BioReagents). cDNA for miRNA and piRNA assays was synthesized from 1.0 μg total RNA using the QuantimiR RT Kit (System Biosciences). cDNA for lncRNA assays was synthesized from 1.0 μg total RNA using Poly(A) Tailing Kit (Ambion) and M-MLV Reverse Transcriptase (Invitrogen) per manufacturer's instructions.

### qRT-PCR

qRT-PCRs were performed using FastStart Universal SYBR Green Master Mix (Roche Diagnostics) and run on a StepOnePlus Real-Time PCR System (Applied Biosystems). Results were analyzed using the ΔΔCt method and normalized to *GAPDH* expression (or *U6* for miRNAs and piRNAs). All primers were obtained from Eurofins Genomics and sequences are listed in Supplementary Table 2. Statistical significance was assessed using Student's *t*-test.

### γ-H2AX Immunofluorescence

UMSCC-10B and HN-30 were transfected with *lnc-JPH1-7* siRNA as previously described and cultured on cover slips. Cells were subsequently treated with 1 μg/mL cisplatin for 24 hours. 48-72 hours after transfection, cells were fixed with 4% paraformaldehyde and blocked in goat serum at room temperature, followed by incubation with anti-phospho-Histone H2A.X (JBW301) mouse monoclonal antibody (Cell Signaling Technology). Cells were then incubated with Alexa Fluor 594 donkey anti-mouse secondary antibody (Life Technologies) and counterstained with Hoechst. Immunofluorescence images were obtained at 63X using a Zeiss inverted fluorescence microscope, and AxioVision 4.8.2 software was used for image capture.

### γ-H2AX foci scoring

γ-H2AX foci were quantified using the FociCounter program (http://focicounter.sourceforge.net/). For each sample, the average number of γ-H2AX foci per cell was determined using at least 7 images taken from different fields on the cover slip (containing ≥40 nuclei total). Statistical significance was assessed using Student's *t*-test.

## SUPPLEMENTARY MATERIALS FIGURES


