# Supplementary material for: The non-coding landscape of head and neck squamous cell carcinoma
Source: Oncotarget. 2016 Jun 13;7(32):51211–22. doi: 10.18632/oncotarget.9979 (PMC5239470; doi:10.18632/oncotarget.9979)
Supplement: Supplementary file 1 [file oncotarget-07-51211-s001.pdf]

The non-coding landscape of head and neck squamous cell carcinoma

Supplementary Materials

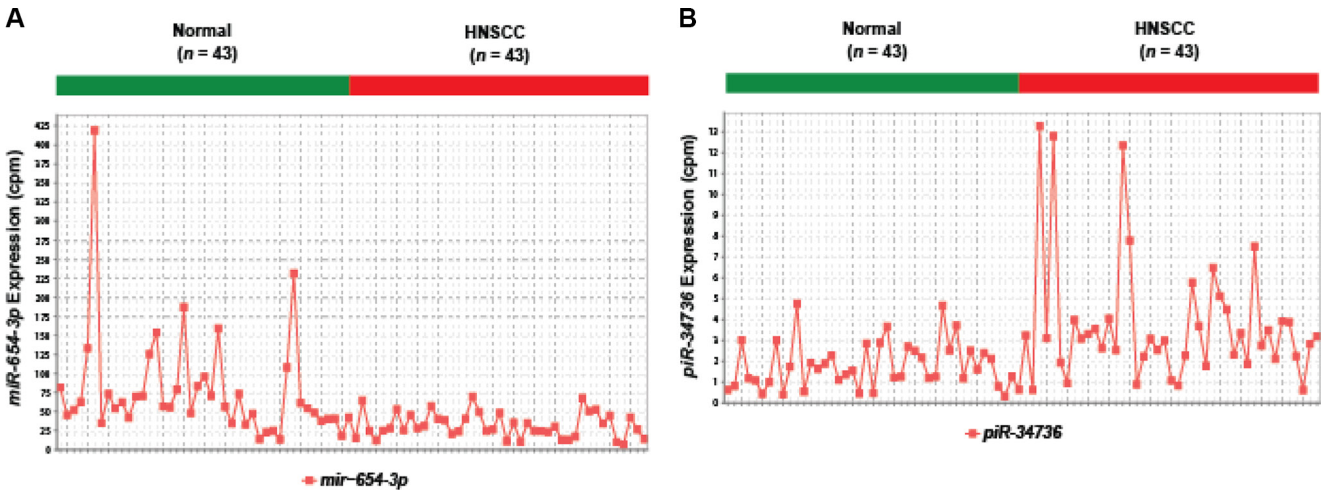

Supplementary Figure S1: Expression plots of selected ncRNAs. (A) *miR-654-3p*, and (B) *piR-34736* in HNSCCs and adjacent normal tissue

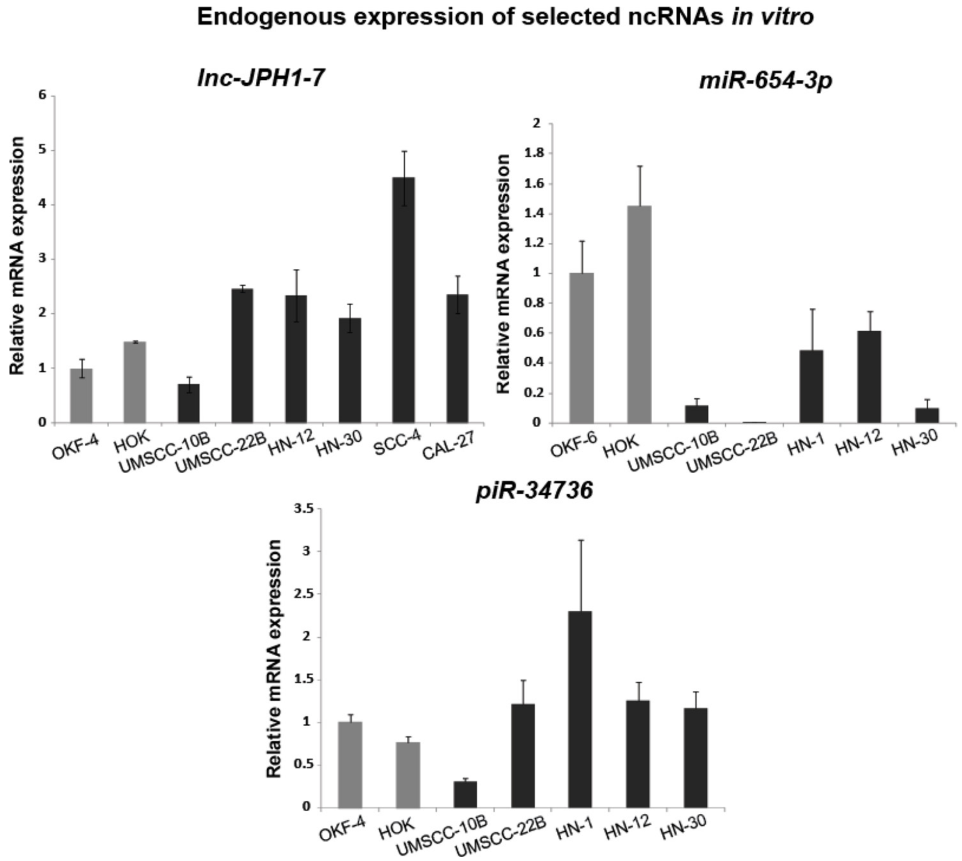

Supplementary Figure S2: Endogenous expression levels of selected ncRNAs in normal epithelial and HNSCC cell lines. OKF-4, OKF-6, and HOK are non-cancerous oral epithelial cell lines; UMSCC-10B, UMSCC-22B, HN-1, HN-2, HN-30, CAL-27, and SCC-4 are established HNSCC cell lines ($n = 6$).

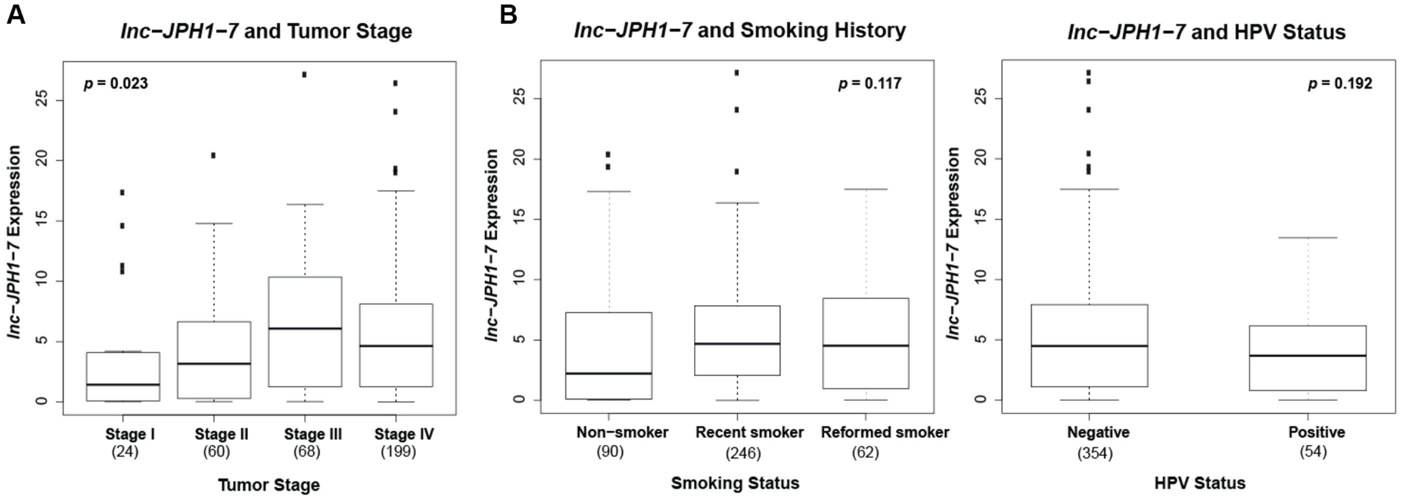

Supplementary Figure S3: Correlation of *lnc-JPH1-7* to additional clinical variables. (**A**) Higher *lnc-JPH1-7* expression is significantly correlated to increasing tumor stage. (**B**) Higher median *lnc-JPH1-7* expression is also observed among patients with history of smoking and HPV-negative patients.

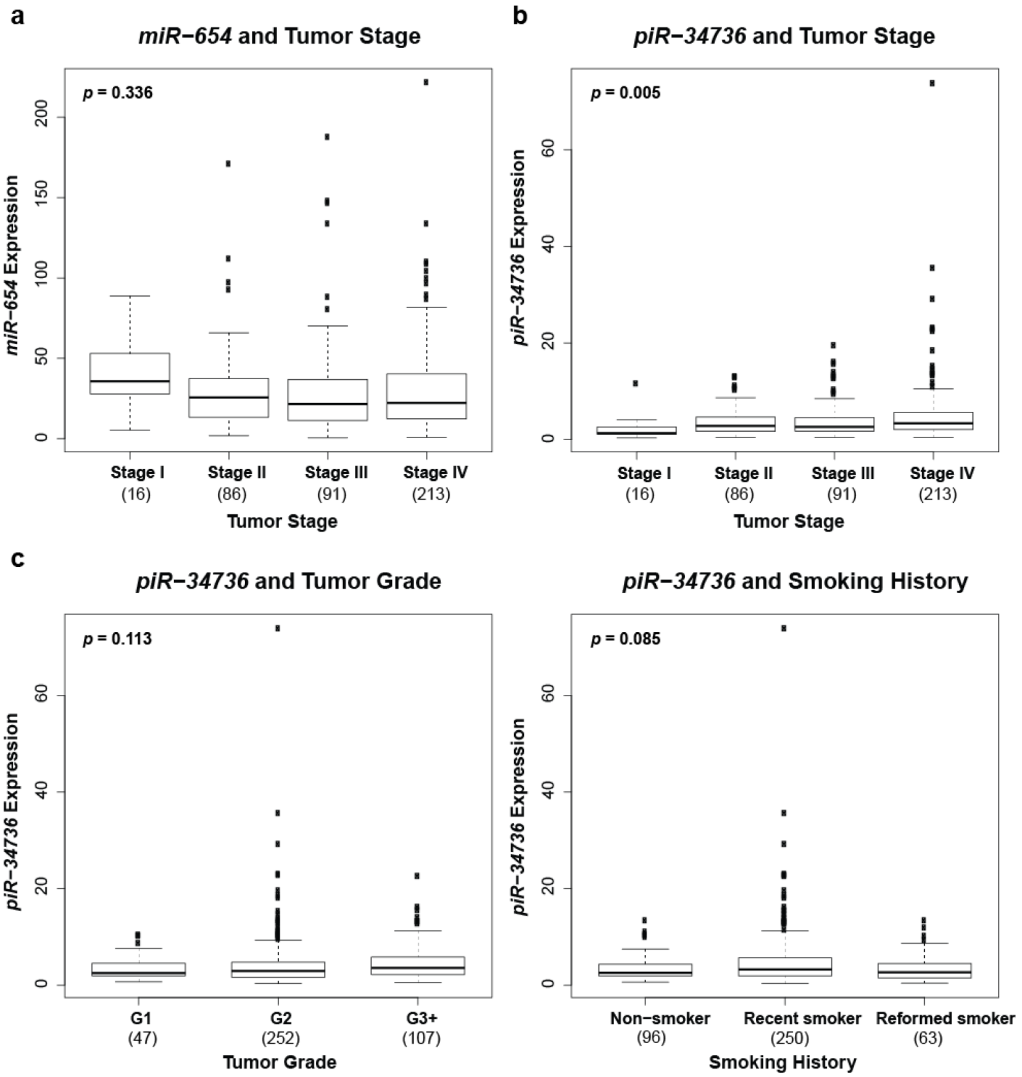

Supplementary Figure S4: Correlation of *miR-654-3p and piR-34736* to additional clinical variables. (**A**) Lower median *miR-654-3p* expression is observed with increasing tumor stage. (**B**) Increasing expression of *piR-34736* is significantly associated with advanced-stage HNSCCs. (**C**) *piR-34736* median levels are elevated in higher grade tumors and among patients with history of smoking.

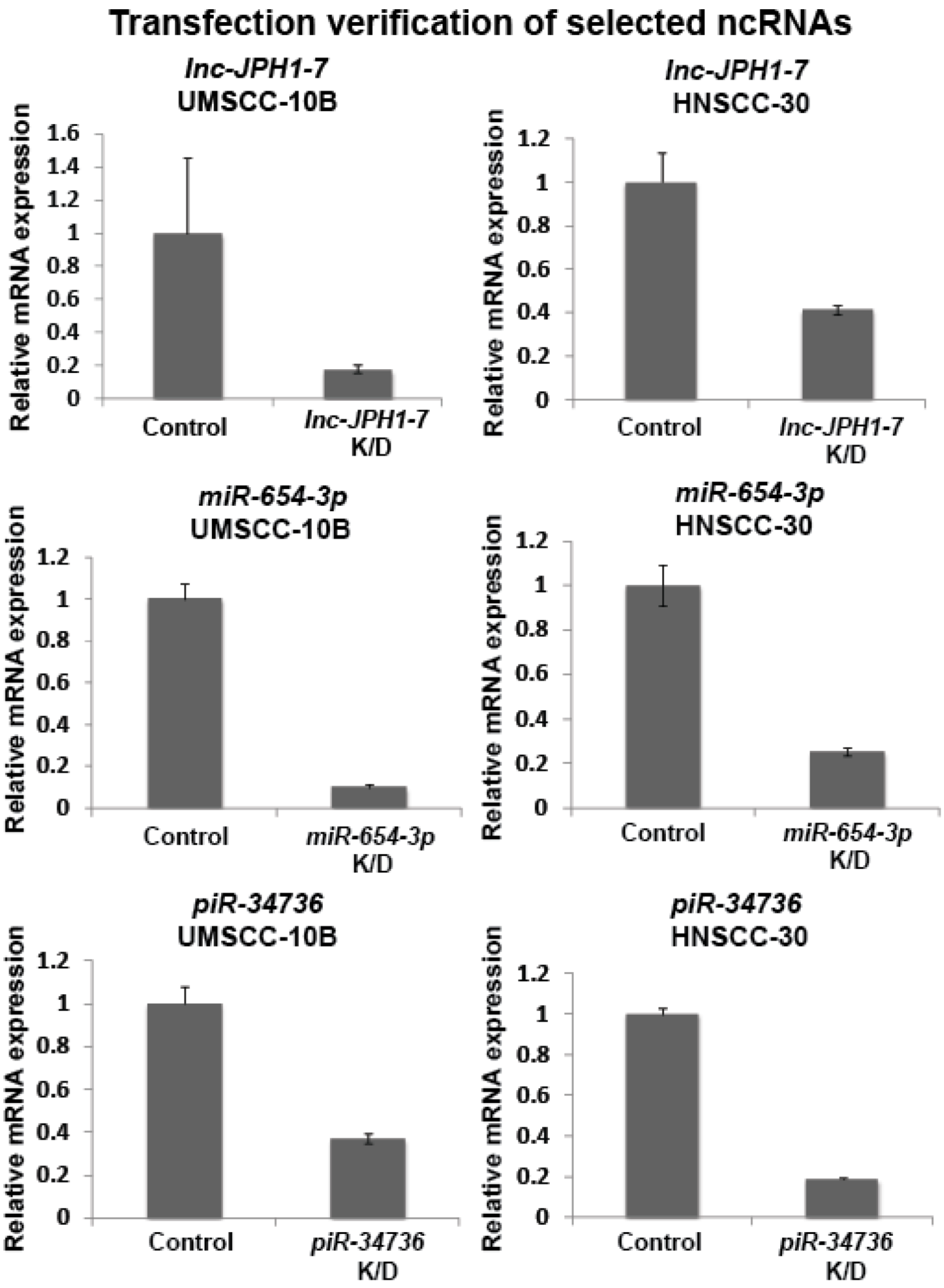

Supplementary Figure S5: Transfection efficiencies of selected ncRNAs in HNSCC cell lines. Verification of successful transfection of siRNA (*lnc-JPH1-7*) and shRNA lentivectors (*piR-34736, miR-654-3p*), in HNSCC cell lines (*n* = 3).

Supplementary Table S1: Demographics and clinical characteristics of TCGA HNSCC cohort (*n* = 422)

Gender	Male	299
	Female	123
Age	< 75 years of age	363
	> = 75 years of age	59
HPV Status	Negative	364
	Positive	58
Tumor Site	Oral cavity	183
	Tongue	132
	Larynx	94
	Pharynx	11
	Missing/Other	2
Tumor Stage	IV	206
	III	71
	II	63
	I	24
	Not Available	58
Tumor Grade	G3+	107
	G2	254
	G1	47
	Not Available	14
Smoking History	Recent Smoker	252
	Reformed Smoker	63
	Lifelong Non-smoker	96
	Not Available	11
Perineural Invasion	Yes	147
	No	150
	Not Available	125

Supplementary Table S2: Sequences of shRNAs and qRT-PCR and expression plasmid primers

A. Primer sequences for qRT-PCRs

Gene	Forward Primer	Reverse Primer
lnc-JPH1-7	5′-GCCACATTCATGGTGTTGAG-3′	5′-CCTGTTTCTTGCCAGGTGTT-3′
miR-654-3p	5′-TATGTCTGCTGACCATCACCTT-3′	--
piR-34736	5′-GAGGAATGATGACAAGAAAAGGCCGAA-3′	--
CDH-1 (E-cadherin)	5′-CTGATGTGAATGACAACGCC-3′	5′-TAGATTCTTGGGTTGGGTCG-3′
VIM (Vimentin)	5′-GGAAATGGCTCGTCACCTTCGT-3′	5′-AGAAATCCTGCTCTCCTCGCCT-3′
SNAI1 (Snail)	5′-CTGCCCTGCGTCTGCGGAAC-3′	5′-GCTTCTCGCCAGTGTGGGTCC-3′
NCAD (N-cadherin)	5′-TGTTTGACTATGAAGGCAGTGG-3′	5′-TCAGTCATCACCTCCACCAT-3′
CYCS (Cytochrome c)	5′-GAGCGAGTTTGGTTGCACTT-3′	5′-TTGCCTCCCTTTTCAACGGT-3′
CASP8 (Caspase-8)	5′-GCAGAGGGAACCTGGTACAT-3′	5′-TCATCCTTGTTGCTTACTTCATAG-3′
CASP9 (Caspase-9)	5′-GCTCTTCCTTTGTTCATCTCC-3′	5′-GTTTTCTAGGGTTGGCTTCG-3′
XIAP	5′-GAGAAGATGACTTTTAACAGTTTTGA-3′	5′-TTTTTTGCTTGAAAGTAATGACTGTGT-3′
BAX	5′-GTTTCATCCAGGATCGAGCAG-3′	5′-CATCTTCTTCCAGATGGTGA-3′
BCL-2	5′-CCTGTGGATGACTGAGTACC-3′	5′-GAGACAGCCAGGAGAAATCA-3′
GAPDH	5′-CTTCGCTCTCTGCTCCTCC-3′	5′-CAATACGACCAAATCCGTTG-3′
U6	5′-GGGGACATCCGATAAAATTGG-3′	5′-ACCATTTCTCGATTTGTGCGT-3′

B. Insert sequences for pmiR-ZIP plasmids

Insert	Forward Primer (BamH1 site/overhang in red, EcoR1 site in blue)	Reverse Primer (BamH1 site in red, EcoR1 site/overhang in blue)
miR-654-3p	5’-GATCCTATGTGTGCTGATCATCACATTCTTCCTG TCAGAAAGGTGATGGTCAGCAGACATATTTTTG-3’	5’-AATTCAAAAATATGTCTGCTGACCATCACCTTTCT GACAGGAAGAATGTGATGATCAGCACACATAG-3’
piR-34736	5’-GATCCAATGATGACGAGACAAGGACGCTTCCT GTCAGACGGCCTTTTCTTGTCATCATTTTTTTG-3’	5’-AATTCAAAAAAATGATGACAAGAAAAGGCCGTC TGACAGGAAGCGTCCTTGTCTCGTCATCATTG-3’
